# Glycolytic metabolism influences global chromatin structure

**DOI:** 10.18632/oncotarget.2929

**Published:** 2015-01-13

**Authors:** Xue-Song Liu, John B. Little, Zhi-Min Yuan

**Affiliations:** Department of Genetics and Complex Diseases, Harvard School of Public Health, Boston, MA 02115, USA

**Keywords:** glycolysis, acetylation, chromatin structure, chemosensitivity

## Abstract

Metabolic rewiring, specifically elevated glycolytic metabolism is a hallmark of cancer. Global chromatin structure regulates gene expression, DNA repair, and also affects cancer progression. But the interrelationship between tumor metabolism and chromatin architecture remain unclear. Here we show that increased glycolysis in cancer cells promotes an open chromatin configuration. Using complementary methods including Micrococcal nuclease (MNase) digestion assay, electron microscope and immunofluorescence staining, we demonstrate that glycolysis inhibition by pharmacological and genetic approaches was associated with induction of compacted chromatin structure. This condensed chromatin status appeared to result chiefly from histone hypoacetylation as restoration of histone acetylation with an HDAC inhibitor reversed the compacted chromatin state. Interestingly, glycolysis inhibition-induced chromatin condensation impeded DNA repair efficiency leading to increased sensitivity of cancer cells to DNA damage drugs, which may represent a novel molecular mechanism that can be exploited for cancer therapy.

## INTRODUCTION

Pathologist have observed for long time that the nucleus of cancer cells show distinct morphological alterations compared to the nucleus of normal cells, and these morphological changes have become parts of the detection standard of cancer [1–3]. Compared to normal cells, cancer cells show large and irregular nuclei, and have small cytoplasmic volume, but the molecular mechanism for these nuclear structure changes is not clear [2].

In the nucleus, the DNA and the histones are arranged in a highly condensed structure known as chromatin. The primary building blocks of chromatin are nucleosomes, which are made up of four core histone proteins (H2A, H2B, H3, and H4), and linker histone H1 [4]. Global chromatin structure regulates many biological processes, including DNA replication, gene transcription, DNA repair [5]. The chromatin structure is highly dynamic, and histone modifications represent an important regulatory mechanism of chromatin structure. For instance, acetylation of lysine residues is associated with histone deposition, chromatin de-condensation and DNA accessibility [6].

Metabolic reprograming is a hallmark of human cancer, accelerated aerobic glycolysis, or Warburg effect, is frequently observed in multiple types of cancer [7]. Tumor cells metabolize most glucose into lactate and thus generate abundant glycolytic intermediates as precursors for macromolecular biosynthesis, which enables tumor cells to meet their increased anabolic and energetic demands due to rapid tumor growth [8]. It was also observed that glycolysis inhibition can sensitize cancer cells to DNA damaging chemo- or radio-therapy agents [9–11], but it was not clear how glycolytic metabolism influence cancer cell sensitivity to these DNA damage agents.

Here we demonstrate that glycolytic metabolism in cancer cells impacts global chromatin structure by controlling histone acetylation, which affects cellular sensitivity to DNA damage by regulating DNA repair efficiency. Glycolysis associated chromatin structure changes may also contribute to the distinct morphological feature of cancer cell nucleus.

## RESULTS

### Glycolytic metabolism stimulates an open global chromatin structure

**Figure 1:**
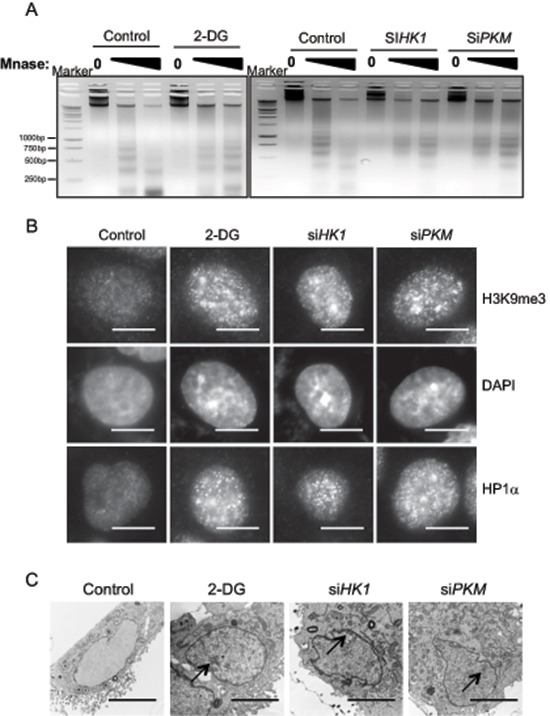
Glycolytic metabolism stimulates open global chromatin structure **(A)** A549 cells treated with glycolysis inhibitor 2-DG or transfected with siRNA targeting key glycolysis enzymes HK1, PKM were digested with increasing concentration of Micrococcal nuclease (MNase). Genomic DNA was analyzed with agarose gel electrophoresis. **(B)** Immunofluorescent staining with heterochromatin marker HP1α and H3K9me3 antibody in A549 cells treated with control, 2-DG or transfected with siRNA targeting HK1, PKM. Scale bar is 5 μm. **(C)** A549 cells treated with 2-DG or transfected with siRNA targeting HK1, PKM were fixed and analyzed by transmission electron microscopy. Arrows indicate heterochromatic regions. Scale bar is 5 μm.

Metabolic reprogramming is a hallmark of cancer, and cancer cells always show elevated glycolytic metabolism [7]. Tumor cells also show discrete changes in their nucleus structure [2]. We examined a potential contribution of tumor metabolism to the distinct chromatin architecture in tumor cells by inhibition of glycolysis. We performed the Micrococcal nuclease (MNase) digestion assay to monitor chromatin structural change in response to glycolysis inhibition. Treatment of human lung carcinoma cell line A549 with 2-Deoxy-D-glucose (2-DG) resulted in increased resistance to the digestion of MNase, indicative of more compact chromatin structure (Figure 1A). To corroborate the result obtained from 2-DG, we used siRNA to knock down the expression of HK1 and PKM, two rate limiting enzymes for glycolysis. Similar to the effect of 2-DG, reduced expression of HK1 and PKM (Supplemental Figure S1) was also associated with induction of MNase resistance in chromatin (Figure 1A). The results together indicate that inhibition of glycolysis in tumor cells caused a change of chromatin structure into a more condensed state.

Condensed chromatin organization usually coincides with a repressive chromatin state, which is characterized by an enrichment of H3K9me3 and heterochromatin protein 1α (HP1α) [5]. We thus carried out immunofluorescence staining of H3K9me3 and HP1 to visualize the alteration of nuclear architecture. Indeed, inhibition of glycolysis by ether 2-DG or knockdown of HK1 or PKM expression resulted in a considerable increase in both H3K9me3 and HP1 staining (Figure 1B). The glycolysis inhibition-induced chromatin condensation was also evident in DAPI staining, which revealed a marked increase of highly dense DAPI-rich structure upon blockage of glycolysis.

To further characterize the interrelationship between glycolysis and chromatin organization, we examined the morphological appearance by transmission electron microscopy (TEM). In line with the more compact chromatin state induced by glycolysis inhibition, cell nuclei imaging by TEM revealed a significant increase in size and abundance of condensed structures along the nuclear envelope and at the nucleolus periphery in cells treated with 2-DG or depleted with HK1 and PKM (Figure 1C). This data provide a further support of an important role for glycolytic metabolism in maintenance of an open chromatin state in cancer cells.

### Glycolytic metabolism is associated with hyper global histone acetylation

**Figure 2:**
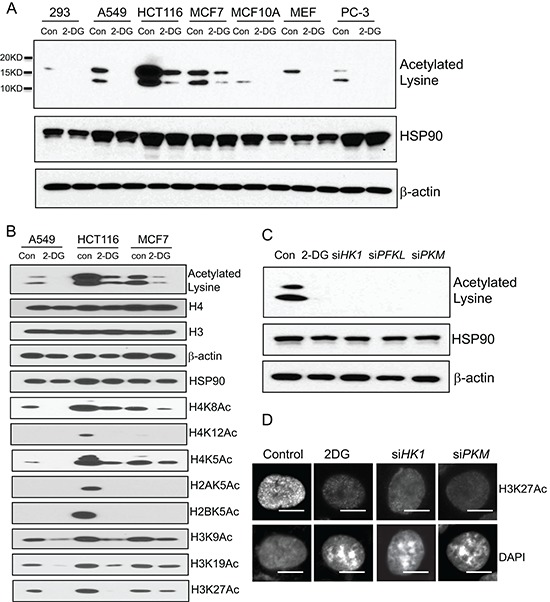
Glycolytic metabolism stimulates global histone acetylation **(A)** A panel of human cell lines (293, A549, HCT116, MCF7, MCF10A, PC-3) and mouse embryonic fibroblast (MEF) were treated with 2-DG (10 mM) for 24 hour. The cells were analyzed by western blot with a lysine acetylation specific antibody. HSP90 and β-actin blots serve as loading controls. **(B)** Individual histone acetylation was probed with specific histone acetylation antibodies. **(C)** A549 cells transfected with siRNA targeting HK1, PFKL, PKM were analyzed by western blot with the lysine acetylation specific antibody. HSP90 and β-actin blots serve as loading controls. **(D)** A549 cells treated with 2-DG, or transfected with siRNA targeting HK1, PKM were subject to immunofluorescence analysis with histone H3K27 acetylation specific antibody. Scale bar is 5 μm.

Having observed the strong impact of cellular glycolysis on chromatin architecture, we sought to understand how chromatin structure was affected by glycolytic metabolism. Chromatin organization can be regulated by multiple mechanisms among which histone post-translational modifications have been well-documented [12]. Histone modifications include acetylation, phosphorylation, methylation, ubiquitylation, sumoylation and poly ADP-ribosylation. Relative to other types of histone modifications that can sometimes have multiple effects, acetylation of lysine residues results in neutralization of the positive charges promoting an open chromatin configuration whereas histone de-acetylation is associated with a condensed compact chromatin structure [12]. Since inhibition of glycolysis in cancer cells induced a more condensed chromatin configuration, we asked whether the transition of chromatin state was accompanied with alteration of histone acetylation. To address this question, we performed immunoblot with a lysine acetylation specific antibody to detect cellular protein acetylation. The antibody recognized two dominant bands with molecular weights approximately 14 and 17KD, and importantly the acetylated bands were significantly decreased by glycolysis inhibitor 2-DG treatment in multiple cell lines (Figure 2A). Based on the protein sizes, the acetylated proteins were predicted to be histones. Consistent with this prediction was the observation that probing with a panel of histone site-specific acetyl antibodies revealed that the acetylation of multiple sites of histones H3, H4, H2A and H2B were considerably decreased upon 2-DG treatment in multiple cell lines (Figure 2B). To complement the results derived from 2-DG treatment, siRNAs targeting key enzymes of glycolysis were employed to inhibit glycolysis, which also led to a marked reduction in global histone acetylation (Figure 2C). In addition, immunofluorescent staining with histone H3K27 acetylation specific antibody revealed strongly decreased histone acetylation upon glycolysis inhibition by 2-DG treatment or siRNA targeting key glycolysis enzymes. In addition, there was a clear inverse correlation between histone acetylation and DAPI-rich structure, consistent with the association of histone acetylation with chromatin organization (Figure 2D). These results implicate a connection of glycolysis with global histone acetylation.

**Figure 3:**
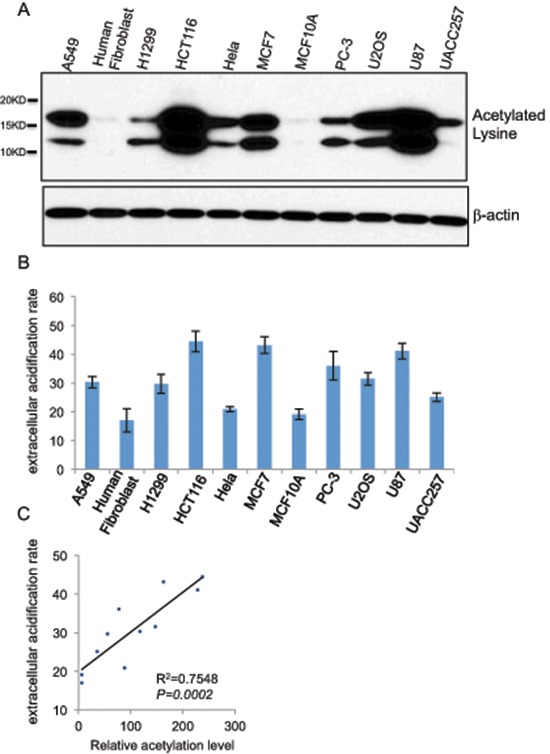
Cellular glycolysis rate positively correlates with the extent of global histone acetylation **(A)** Global histone acetylation levels of 11 different human cell lines (A549, human fibroblast, H1299, HCT116, HeLa, MCF7, MCF10A, PC-3, U2OS, U87, UACC257) were determined by western blot with a lysine acetylation specific antibody with β-actin as a loading control. **(B)** Glycolytic rates shown as the extracellular acidification rates (ECAR) of 11 cell lines were measured by seahorse XF^e^ Extracellular Flux Analyzers, Data shown are average values of three experiments with error bar indicate mean ± s.d. **(C)** The correlation between the glycolytic rate and global histone acetylation level in 11 human cells.

While different cell lines exhibited invariably reduced histone acetylation upon glycolysis inhibition we noted an apparent difference in the basal level of histone acetylation. Given the observed close correlation of glycolysis and histone acetylation, we asked whether the different basal level of histone acetylation might reflect a different rate of glycolysis. We addressed this question by comparing the cell lines for the correlation of histone acetylation with the rate of glycolysis, Immunoblot with the acetyl-specific antibody revealed that among 11 human cell lines tested, the level of acetylated histones in HCT116, U87 was the highest, while human fibroblast and MCF10A exhibited the lowest extent of histone acetylation (Figure 3A). We analyzed the rate of glycolysis with a seahorse XF^e^ Extracellular Flux Analyzer that monitors the extracellular acidification rate (ECAR) to reflect the kinetics of glycolysis. Indeed, the 11 human cell lines displayed different rates of glycolysis (Figure 3B). Remarkably, the cellular glycolysis rate positively correlated with histone acetylation levels (Figure 3C). In line with the notion that the elevated glycolysis is a hallmark of cancer cells, non-transformed cells, MCF-10A and human fibroblasts exhibited a markedly lower rate of glycolysis that corresponded with a lower extent of histone acetylation.

### Essential role of histone acetylation in regulating chromatin structure

**Figure 4:**
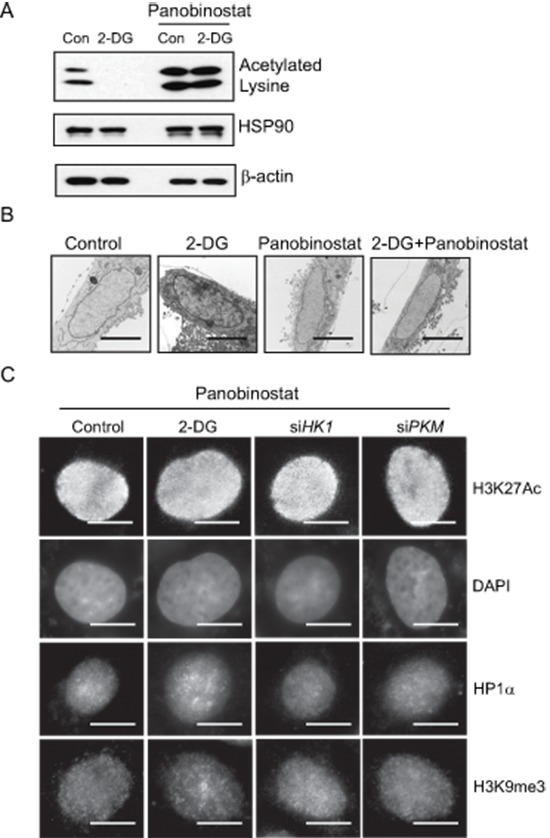
Role of histone acetylation in regulating glycolysis induced chromatin structure changes **(A)** HDAC inhibitor panobinostat (80 nM) was added to cells treated with or without 2-DG. Histone acetylation was detected by immunoblot with a lysine acetylation specific antibody. HSP90 and β-actin blots serve as loading controls. **(B)** Transmission electron microscopy analysis was performed in A549 cells treated with control, 2-DG (10 mM), panobinostat (80 nM) or in combination. Scale bar is 5 μm. **(C)** Immunofluorescent staining with H3K27Ac, or heterochromatin marker HP1α and H3K9me3 antibody in A549 cells treated with 2-DG or transfected with siRNA targeting *HK1*, *PKM* in the presence of panobinostat (80 nM). Scale bar is 5 μm.

Given that inhibition of glycolysis was associated with induction of histone de-acetylation as well as condensed chromatin configuration, we wondered whether glycolytic metabolism might affect chromatin organization via modulation of histone acetylation. Treatment with a HDAC inhibitor panobinostat (80 nM, 24 hour) blocked the decrease of histone acetylation in 2-DG-treated cells (Figure 4A), resulting in almost complete restoration of the level of histone acetylation. We went on testing the effect of this HDAC inhibitor on glycolysis inhibition-induced change in chromatin structure. TEM analysis of cell nuclei showed that the condensed nuclear structures in 2-DG-treated cells were substantially diminished by addition of the HDAC inhibitor (Figure 4B). Immunofluorescence staining further confirmed that glycolytic metabolism regulated chromatin organization via modulation of histone acetylation. As shown in Figure 4C, glycolysis inhibition-induced heterochromatin like structure such as DAPI-rich, HP1 and H3K9me3 staining was abrogated by the treatment with the HDAC inhibitor. The results together implicate that elevated glycolysis in cancer cells enhances histone acetylation leading to an open chromatin state.

### Role of HAT and HDAC in glycolysis regulated histone acetylation

**Figure 5:**
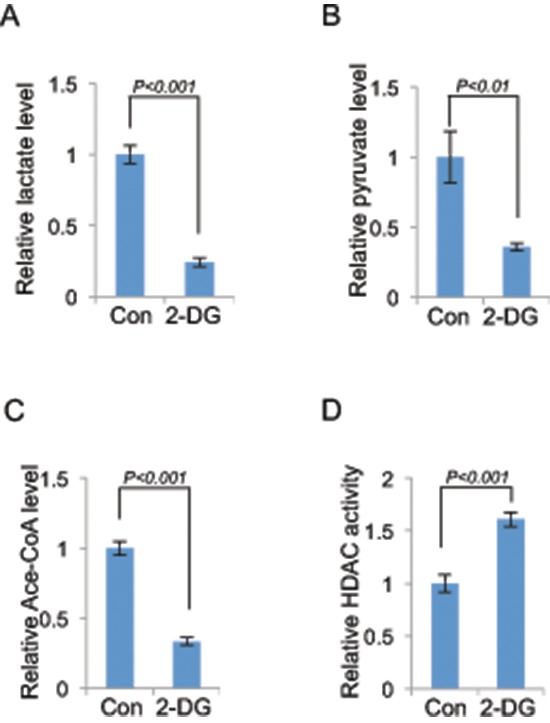
Both HDAC and HAT are involved in glycolysis induced histone acetylation The level of lactate **(A)**, pyruvate **(B)**, acetyl-CoA **(C)** or HDAC activity **(D)** in 2-DG treated (10 mM, 24 h) or control A549 cells was measured. Data shown are average values of three experiments with error bar indicate mean ± s.d.

The steady state of histone acetylation is determined by a balanced action of histone acetyltransferase (HAT) and histone deacetylase (HDAC) [6]. The reduced histone acetylation by glycolysis inhibition could result from a decreased activity of HAT or increased activity of HDAC. It has been reported that metabolites or intermediates of the glycolytic pathway contribute to histone modifications, for instance, Acetyl-CoA, which provides the acetyl group required for the acetylation reaction, stimulates histone acetylation [13]. Pyruvate and lactate promote histone acetylation by inhibiting the activity of HDAC [14, 15]. We thus investigated the molecular mechanism underlying glycolysis-mediated modulation of histone acetylation by measuring the abundance of glycolytic metabolites. The result revealed that inhibition of glycolysis with 2-DG resulted in significant reduction of lactate, pyruvate and acetyl-CoA abundance (Figure 5A–5C). In addition, HDAC activity was found elevated in 2-DG-treated cells (Figure 5D). The results together suggest that glycolysis regulates histone acetylation via modulation of the activity of both HAT and HDAC. We also made an attempt to identify HDACs that were involved in glycolysis-mediated histone acetylation. To achieve this, we knocked down the expression of eleven HDACs individually or in combination. Results indicated that knockdown of multiple HDACs alleviated albeit partially the effect of 2-DG on global acetylation (Supplemental Figure S2), suggesting an involvement of multiple HDACs, which is consistent with previous reports showing that glycolytic metabolites were able to interfere with the activity of multiple HDACs [13, 14]. Knockdown of HDACs 3, 4 and 1/2/3/8 mix were unexpectedly associated with reduction of basal histone acetylation (Supplemental Figure S2), likely due to cellular toxicity.

### Glycolysis confer efficient DNA repair, and chromatin structure alteration is involved in this efficient DNA repair

**Figure 6:**
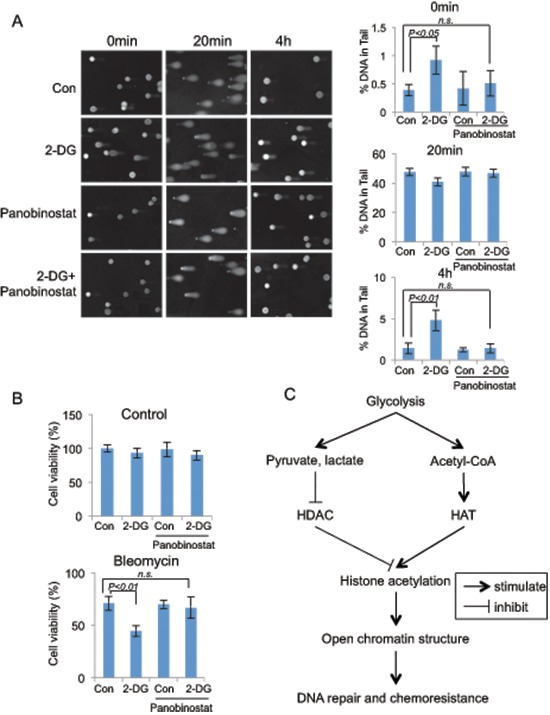
Glycolysis induced chromatin structure change affects DNA repair efficiency and chemo-sensitivity **(A)** Comet assay were performed in A549 cells that were treated with glycolysis inhibitor 2-DG (10 mM, 24 hours), HDAC inhibitor panobinostat (80 nM, 24 hours) or combined as indicated, DNA damage were induced by bleomycin (40 μM) treatment for 20 min at 37°C. Bleomycin was then washed away and the cells were cultured in medium with the indicated chemicals for 4 hours. DNA damage levels were shown as % of DNA in comet tail. Error bars represent mean ± s.e.m. **(B)** Glycolysis inhibitor 2-DG sensitize A549 cells to DNA damage drugs. A549 cells were treated with control or 2-DG, bleomycin or combination for 24 hours. Quantified cell viabilities of three independent experiments are shown as mean ± s.d. **(C)** Proposed model for glycolysis regulated global chromatin structure changes. Glycolysis stimulates global histone acetylation by repressing HDAC through increased production of pyruvate, lactate; and by stimulating HAT activity via increased cellular concentration of Acetyl-CoA. Increased global histone acetylation stimulates the open chromatin structure, which contributes to the efficient DNA repair, and chemoresistance.

Chromatin structure plays an important role in regulation of nuclear processes including DNA repair, which is usually initiated by active recruitment of components of DNA repair machinery to the site of DNA lesion [16–18]. Compact chromatin structure can hinder the access of the DNA repair machinery and thus impede the efficiency of DNA repair. We hypothesized that glycolytic metabolism might affect DNA repair via regulation of chromatin organization. We tested the hypothesis by measuring DNA repair efficiency in cells treated with or without 2-DG using comet assay. Interestingly, treatment of cells with 2-DG was associated with a slight induction of comet tail even in the absence of any DNA damage agent (Figure 6A), suggesting that condensed chromatin structure negatively affected the basal DNA repair process. Bleomycin, a chemical known to cause DNA double strand break, was used to induce DNA damage. As expected, treatment of cells with bleomycin induced a dramatic increase in the comet tail length at 20 min (Figure 6A). The comet tails were almost completely disappeared by 4 h post-treatment, reflecting the process of DNA repair. Of note, there was a considerable comet tails remained at 4 h in 2-DG-treated cells, suggesting an impairment of DNA repair by glycolysis inhibition (Figure 6A). To examine whether this attenuated DNA repair was caused by condensed DNA structure due to histone deacetylation, we induced restoration of histone acetylation by treating cells with the HDAC inhibitor. Remarkably, treatment of cells with the HDAC inhibitor completely rescued the efficiency of DNA repair (Figure 6A). The data together support a critical importance of an open chromatin configuration for effective DNA repair.

DNA damage, if unrepaired, can serve as a signal triggering cell death [19]. The compromised efficacy of DNA repair in 2-DG-treated cancer cells would predict an increased sensitivity to DNA damage agents. We tested this by treating cells with a combination of 2-DG with DNA damage drug bleomycin. Indeed, 2-DG treatment significantly heightened the sensitivity of cancer cells to bleomycin-induced killing and importantly, restoration of histone acetylation by inhibition of HDAC abolished the effect of 2-DG (Figure 6B), implicating that 2-DG sensitized cancer cells to the DNA damage agent via inducing decreased histone acetylation and subsequent condensed chromatin structure. In contrast with cancer cells, non-cancerous cells would be expected to be less sensitive to the sensitizing effect of glycolysis inhibition because of their low level of glycolytic metabolism. We tested this possibility by comparing MCF-7 with MCF-10A for their response to 2-DG. In line with the difference in the rate of glycolysis, 2-DG sensitized glycolytic MCF-7 cells but not MCF-10A cells to bleomycin-induced cell death (Supplemental Figure S3).

## DISCUSSION

In this study, we provide multiple lines of evidence connecting tumor metabolism with chromatin organization. Glycolytic metabolism stimulates an open chromatin structure by promoting global histone acetylation, a consequence of the glycolytic metabolites or intermediates-mediated HDAC inhibition and HAT stimulation. Data from RNAi-mediated knockdown experiments suggest an involvement of multiple HDACs in glycolysis induced histone acetylation (Supplemental Figure S2), which is consistent with the previous reports that the glycolytic metabolites were able to interfere with the enzymatic activity of several HDACs [13, 14]. One of the biological outcomes associated with glycolysis-induced open chromatin structure is an efficient DNA repair, which renders cancer cells resistance to DNA damage therapeutic drugs (Figure 6C).

Elevated glycolysis is commonly observed in human cancer cells. Oncogenic pathways have been shown to contribute to this metabolic adaptation of neoplastic cells [7]. Well-known examples include that oncogenic Akt stimulates aerobic glycolysis [21]. While responsible for increased glutaminolysis, Myc also promotes glycolysis through regulating directly or indirectly the expression of glycolytic genes [22]. The mammalian target of rapamycin complex 1 (mTORC1) enhance glycolysis by stimulating the expression of hypoxia-inducible factor (HIF1a), a master transcriptional regulator of glycolysis genes [23, 24]. It is well known that elevated glycolysis can provide metabolic intermediates for synthetic pathways, critical for cancer cell proliferation and survival [8]. It is, however, less clear whether and how glycolysis influence cancer cell sensitivity to DNA damage agents. Increased activity of DNA repair is one of the major mechanisms for cancer cells to acquire resistance to DNA damage agents. Global chromatin structure affects DNA repair efficiency in that an open chromatin structure can facilitate DNA repair likely through an efficient recruitment of DNA repair machinery [16]. Indeed, glycolysis inhibition-induced chromatin condensation impeded DNA repair in cancer cells, resulting in a heightened sensitivity to DNA damaging agent. Because of the difference in metabolism, non-cancerous cells such as MCF-10A exhibited little sensitivity to 2-DG. This differential sensitivity of cancer cells and non-cancerous cells to glycolysis inhibition may create a unique therapeutic opportunity to preferentially sensitize cancer cells to DNA damage drugs with inhibitors of glycolysis.

It was reported that global histone acetylation patterns could serve as a prognostic factor for multiple cancers [25, 26], for instance, H3K18 and H4K12 acetylation were positively correlated with increased prostate grade, and increased acetylation predicted poor prognosis [25]. But in these previous reports, it was not known how histone acetylation predict cancer patients prognosis. Our work suggests that the increased H3K18 and H4K12 acetylation may be caused by elevated glycolysis, which contributes to tumor progression and poor outcome [7]. While further confirmation is necessary, work from a study in yeast suggested GCN5 as a potential HAT responsible for glycolysis-induced histone acetylation at multiple sites [20]. Glycolysis associated chromatin structure alterations may also contribute to the distinct nuclear morphological changes found in cancer cells, a feature often used by pathologist to identify malignant cells.

In conclusion, the data presented in this report demonstrate that glycolysis inhibition markedly influences global chromatin structure by down regulating global histone acetylation. The glycolysis associated chromatin structure changes affects DNA accessibility, DNA repair and cellular sensitivity to DNA damage agents.

## MATERIALS AND METHODS

### Cell culture

Human lung carcinoma cell line A549 and H1299; Human melanoma cell line UACC257 were cultured in RPMI (Corning, Cellgro) plus 10% Fetal Bovine Serum (FBS) (Gibco), 100 U/ml penicillin G and 100 μg/ml streptomycin (Corning, Cellgro). Human cervix adenocarcinoma cell line HeLa; Human prostate adenocarcinoma cell line PC-3; Normal Human fibroblast; Mouse embryonic fibroblast (MEF); Human Embryonic Kidney 293 cells; Human mammary gland adenocarcinoma cell line MCF7; Human bone osteosarcoma cell line U2OS were cultured in DMEM (Corning, Cellgro) plus 10% FBS, Penicillin-Streptomycin antibiotics. Human mammary epithelial cell line MCF10A were cultured in DMEM/F-12 (Corning, Cellgro) plus 5% horse serum (Invitrogen), EGF (20ng/ml, Peprotech), Hydrocortisone (500ng/ml, Sigma), cholera toxin (100ng/ml, Sigma), Insulin (10ug/ml, Sigma), Penicillin-Streptomycin antibiotics. Human glioblastoma cell line U87 were cultured in EMEM (Corning, Cellgro) plus 10% FBS, Penicillin-Streptomycin antibiotics. Human colorectal carcinoma cell line HCT116 were cultured in McCoy's 5A (Corning, Cellgro) plus 10% FBS, Penicillin-Streptomycin antibiotics. All cells were cultured in 37°C, 5% CO2 incubator.

### Antibodies and reagents

H3K9me3 antibody (Millipore, #07–442); HP1α antibody (cell signaling, #2616); Acetylated-Lysine (Ac-K2–100) antibody (Cell Signaling, #9814); Histone H3 antibody (Cell Signaling, #4499); Histone H4 antibody (Cell Signaling, #2935); H2AK5 antibody (Cell Signaling, #2576); H2BK5 antibody (Cell Signaling, #12799); H3K9Ac antibody (Cell Signaling, #9649); H3K14Ac antibody (Cell Signaling, #7627); H3K27Ac antibody (Cell Signaling, #8173); H3K56Ac antibody (Cell Signaling, #4243); H4K5Ac antibody (Cell Signaling, #8647); H4K8Ac antibody (Cell Signaling, #2594); H4K12Ac antibody (Cell Signaling, #2591); Anti-β-actin antibody (AC-15, Sigma); Anti-Hsp90 antibody (AC-16, Sigma) was used for western blot; 2-Deoxy-D-glucose (2-DG), bleomycin was brought from Sigma; HDAC inhibitor Panobinostat was purchased from LC Laboratories.

### MNase assay

MNase accessibility assay was performed as described [27]. Cells (6-well plates, 70% confluency) were permeabilized with 0.025% lysolecithin (Sigma) in permeabilization buffer (150 mM sucrose; 80 mM KCl; 35 mM HEPES, PH7.4; 5 mM K_2_HPO_4_; 5 mM MgCl_2_; 0.5 mM CaCl_2_) for 2 minutes at room temperature. The cells were then digested with indicated amount of MNase (NEB) in digestion buffer (150 mM sucrose; 50 mM Tris, PH 7.5; 50 mM NaCl; 2 mM CaCl_2_) for 5 minutes at room temperature. The digestion was stopped with lysis buffer (20 mM Tris, PH7.4; 200 mM NaCl; 2 mM EDTA; 2% SDS; 0.2 mg/ml proteinase K; 0.2mg/ml RNase A). DNA was purified by Phenol–chloroform extraction and ethanol precipitation. 500 ng DNA was loaded in agarose gel, stained with ethium bromide.

### Immunofluorescence

Cells were seeded in 24-well plates containing round glass coverslips at the density of 2×104/well. 24 hours after plating, cells were fixed with paraformaldehyde, permeabilized in 0.1% Triton-X-100/phosphate buffered sulphate (PBS). Coverslips were then incubated with primary antibody diluted in 1% BSA/PBS for 1 h. After washing, coverslips were incubated in secondary antibody diluted in 1% BSA/PBS for 1 h. Coverslips were washed, stained with DAPI, mounted and analyzed by confocal microscopy (Zeiss).

### Immunoblot

Cells were lysed in buffer (50 mM Tris, pH8.0; 150 mM NaCl; 0.5% NP-40; 0.2% SDS and protease inhibitor cocktail (Roche)). Protein concentrations of the lysates were measured by Bradford assay. The lysates were then resolved by SDS–PAGE and immune-blotted with the indicated antibodies. The immune blot signals were quantified by Image J software.

### Transmission electron microscope

Cells was fixed by adding 2X fixative directly to the medium, the final concentration of fixative contain 1.25% formaldehyde, 2.5% glutaraldehyde, 0.03% picric acid, 0.1 M Sodium cacodylate buffer, pH 7.4. The cells were embedded in Epon, stained with Uranyl Acetate. The sections (80 nm) were viewed with a Tecnai G^2^ Spirit Bio TWIN electron microscope at the core facility of Harvard Medical School.

### Comet assay

DNA lesions were assessed using a single-cell gel electrophoretic comet assay kit (Trevigen). Cells were combined with low melting point agarose and pipetted onto a slide. The cells were lysed, then subject to electrophoresis at 20 volts for 30 minutes in TBE buffer. Following electrophoresis, slides were washed, dehydrated and stained with SYBR Green I. Images were taken with a fluorescent microscope and scored by CometScoreTM software (TriTek Corporation). The percentage of DNA in comet tails is scored from 200 cells of 3 different experiments, and shown as mean ± s.e.m.

### HDAC activity assay

Whole cell lysates (extraction buffer 50 mM HEPES PH8.0; 0.5 mM EDTA; 0.1 mM EGTA; 420 mM NaCl; 0.5% NP40; 10% glycerol) were extracted from same number of A549 cells treated with control or 2-DG (10 mM, 24 hours). HDAC activity in the total cell lysates were measured using a HDAC Activity Colorimetric Assay Kit (Biovision) according to the attached protocol.

### Acetyl-CoA, pyruvate and lactate concentration measurement

Total cell lysates were extracted from same number of A549 cells treated with control or 2-DG (10 mM, 24 hours). Proteins in the lysates were removed by 10KD molecular weight spin filter (BioVision). Acetyl-CoA, pyruvate and lactate concentration in the samples were measured with PicoProbe™ Acetyl-CoA Fluorometric Assay Kit (BioVision), Pyruvate Colorimetric/Fluorometric Assay Kit (BioVision), Lactate Colorimetric/Fluorometric Assay Kit (BioVision) respectively.

### Cell viability assay

A549, MCF7, MCF10A cells were seeded in 96 well plates, 12 hours later cells were treated with 2-DG (2 mM), bleomycin (40 μM), panobinostat (80 nM) or combination as indicated in the figure legend for 24 hours. The numbers of viable cells in culture were quantified using CellTiter-Glo^®^ Luminescent Cell Viability Assay kit (Promega) following attached protocol.

### Statistical analysis

Unpaired Student's *t*-test was used for the comparisons of the means, error bars represent standard deviation (s.d.), unless otherwise stated. The statistical significances between data sets were expressed as *p* values, and *p* < 0.05 was considered statistically significant.

## SUPPLEMENTARY METHODS FIGURES


