# Supplementary material for: Glycolytic metabolism influences global chromatin structure
Source: Oncotarget. 2015 Jan 13;6(6):4214–25. doi: 10.18632/oncotarget.2929 (PMC4414184; doi:10.18632/oncotarget.2929)
Supplement: Supplementary file 1 [file oncotarget-06-4214-s001.pdf]

SUPPLEMENTARY METHODS

siRNA transfection

siRNA targeting human *HK1, PFKL, PKM, HDAC1–11* was synthesized by Sigma. Sequences of these siRNA: siHK1: CACAGUAUU CCCGGC GUUU; siPFKL: CACAUGACG GAGAAGAUGA; siPKM: CGUGGAUGA UGGGCU UAUU; siHDAC1: GUUAGG UUGCUU CAAUCUA; siHDAC2: GUGUAA UGACGG UAUCAUU; siHDAC3: GAUCGA UUGGGC UGCUUUA; siHDAC4: CAGCCAAG CUUCUGCA GCA; siHDAC5: GACUGUUA UUAGCACC UUU; siHDAC6: CACUGAUC AGGCCAUA UUU; siHDAC7: CAUGUUUC UGCCAAAU GUU; siHDAC8: CCUUCAAU GGCAGUUG GCA; siHDAC9: CACUUCAU UCAAGAAG CGA; siHDAC10: GCCUUUCC UGCGAGAG UCA; siHDAC11: GACUAGGC CUGC AUCC UAA; For siRNA transfection, 3x10^5 cells were reverse transfected with a mixture of siRNA and Lipofectamine® RNAiMAX Reagent (Invitrogen) in 35cm dish according to manufacture`s protocol.

Real-time PCR to quantify gene expression

Total RNA was extracted with TRIzol® Reagents (Invitrogen) according to the provided protocol. 1 µg total RNA was reversed transcribed with iScript™ cDNA Synthesis Kit (Bio-Rad). Real time quantitative PCR was performed using diluted cDNA, SYBR® Green JumpStart™ Taq ReadyMix (Sigma) and appropriate primers in StepOnePlus Real Time PCR System (Applied Biosystems). 18s was used as an endogenous control for normalization. Primer sequence for the following genes:

Hu18s-5:CAGCCACCCGAGATTGAGCA; Hu18s-3:TAGTAGCGACGGGCGGTGTG; HDAC1rt-5:CGC CCTCACAAAGCCAATG; HDAC1rt-3:CTGCTTGCT GTACTCCGACA; HDAC2rt-5:ATGGCGTACAGTCA AGGAGG; HDAC2rt-3:TGCGGATTCTATGAGGC TTCA; HDAC3rt-5:GCAAGGCTTCACCAAGAGTCT; HDAC3rt-3:AGATGCGCCTGTGTAACGC; HDAC4rt-5:CCTGGGAATGTACGACGCC; HDAC4rt-3:CCCG TCTTTCCTGCGTAAC; HDAC5rt-5:TCTTGTCGAAG TCAAAGGAGC; HDAC5rt-3:GAGGGGAACTCTG GTCCAAAG; HDAC6rt-5: ACCCCAGTGTCCTCTA TTTCTC; HDAC6rt-3:CCTGGTTCCAAGGCACA TTGA; HDAC7rt-5:TGCCCAGTCCTTAATGACCAC; HDAC7rt-3: CACCTGGACGTGAGTTTTGAG; HDAC8rt-5:TCGCTGGTCCCGGTTTATATC; HDAC8rt-3:TACTGGCCCGTTTGGGGAT; HDAC9rt-5 AGTAG AGAGGCATCGCAGAGA; HDAC9rt-3 GGAGTGTCT TTCGTTGCTGAT; HDAC10rt-5:CAGTTCGACGCC ATCTACTTC; HDAC10rt-3: CAAGCCCATTTTGCA CAGCTC; HDAC11rt-5:ATCTACCCTGGGGATC GCTTT; HDAC11rt-3 CTCCTGACATTCCTCTCCACC.

SUPPLEMENTARY FIGURES

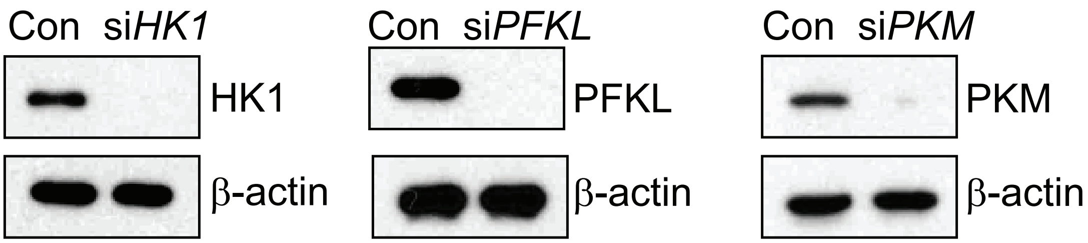

Supplementary Figure S1: Immunoblot blot was carried out with the indicated antibodies to determine the knockdown efficiency of si*HK1*, si*PFKL* and si*PKM*. β-actin serves as the loading control.

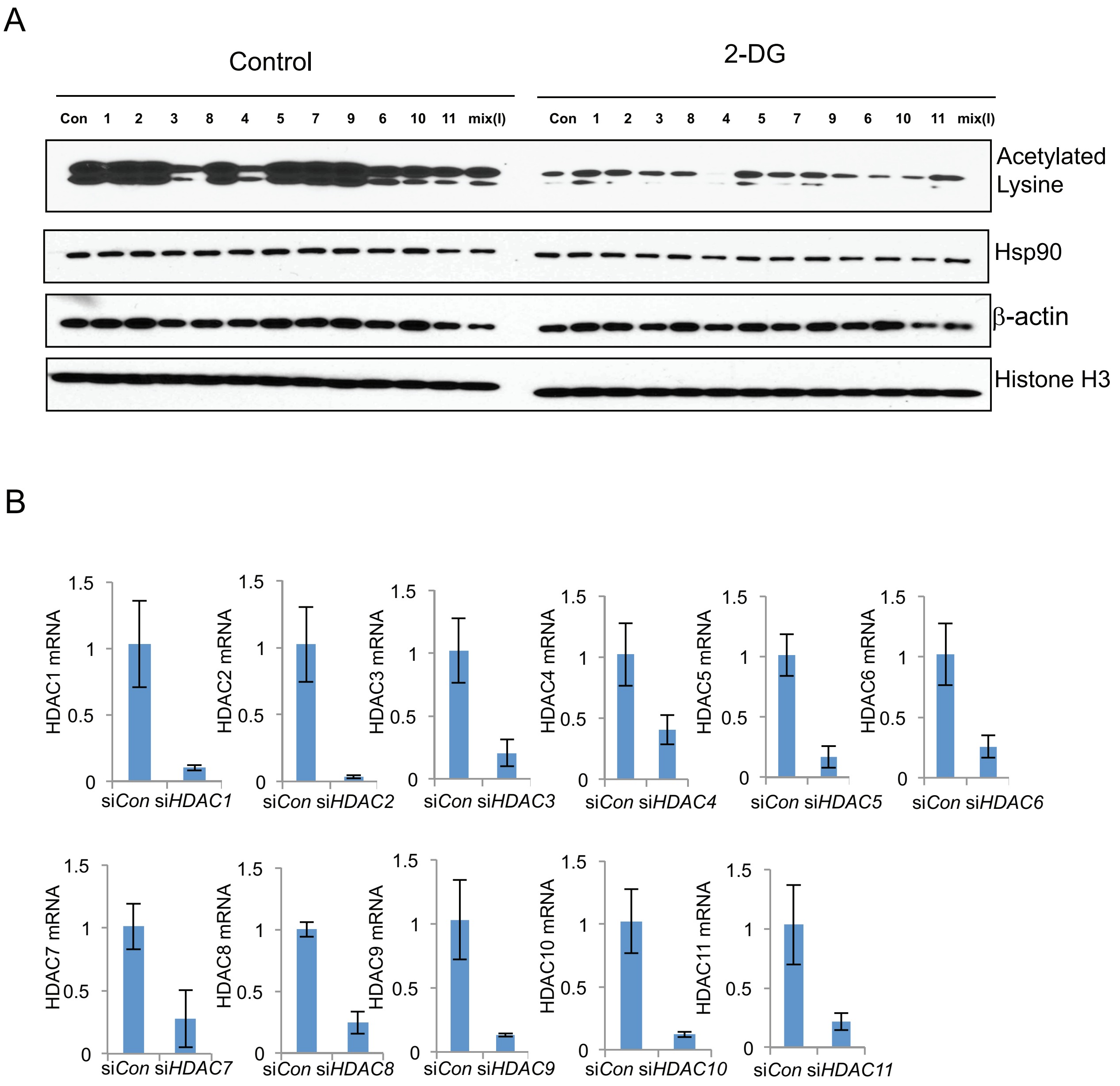

Supplementary Figure S2: Multiple HDACs are involved in glycolysis associated histone acetylation. (A) A549 cells were treated with control, 2-DG, siRNA targeting HDAC1 to HDAC11, or combination. Mix siRNA contain siHDAC1, 2, 3, 8. Acetylation was detected by Western blot. HSP90, β-actin, Histone 3 served as loading control. **(B)** Real time PCR was performed to quantify the knockdown efficiency of siRNA targeting *HDAC1* to *HDAC11*.

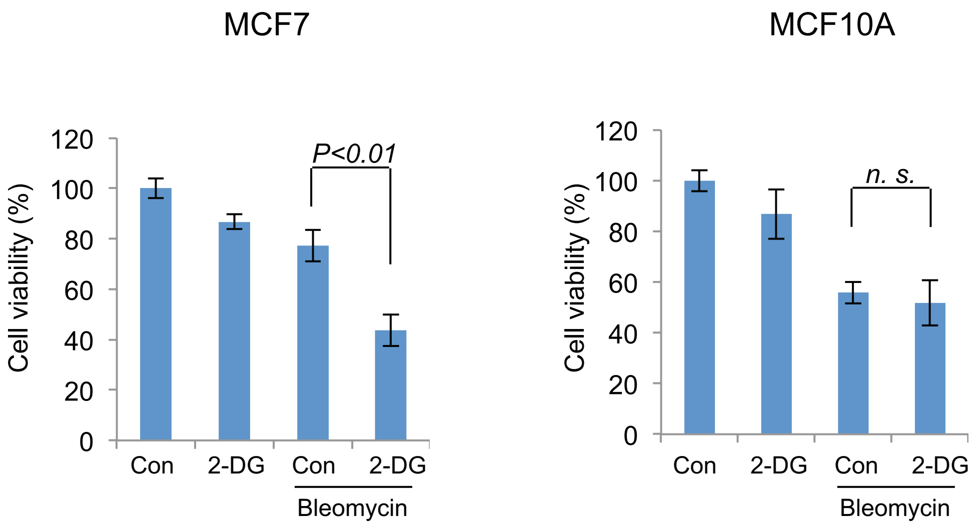

Supplementary Figure S3: Glycolysis status determines cellular sensitivity to combined 2-DG and bleomycin treatment. MCF7 and MCF10A were treated with or without 2-DG followed by bleomycin treatment. Cell viability was determined and quantified. The numbers are mean ± s.d. from three experiments.
